# Intermediate Filaments and Polarization in the Intestinal Epithelium

**DOI:** 10.3390/cells5030032

**Published:** 2016-07-15

**Authors:** Richard A. Coch, Rudolf E. Leube

**Affiliations:** Institute of Molecular and Cellular Anatomy, RWTH Aachen University, Wendlingweg 2, Aachen 52074, Germany; rcoch@ukaachen.de

**Keywords:** keratin, cytokeratin, endotube, brush border, *C. elegans* apical junction, desmosome, γ-tubulin ring complex, atypical protein kinase C, PAR proteins

## Abstract

The cytoplasmic intermediate filament cytoskeleton provides a tissue-specific three-dimensional scaffolding with unique context-dependent organizational features. This is particularly apparent in the intestinal epithelium, in which the intermediate filament network is localized below the apical terminal web region and is anchored to the apical junction complex. This arrangement is conserved from the nematode *Caenorhabditis elegans* to humans. The review summarizes compositional, morphological and functional features of the polarized intermediate filament cytoskeleton in intestinal cells of nematodes and mammals. We emphasize the cross talk of intermediate filaments with the actin- and tubulin-based cytoskeleton. Possible links of the intermediate filament system to the distribution of apical membrane proteins and the cell polarity complex are highlighted. Finally, we discuss how these properties relate to the establishment and maintenance of polarity in the intestine.

## 1. Introduction

The shape of a cell and its subcellular organization are mainly determined by the cytoskeleton, which is composed of three major filament systems, each with distinct structural and functional signet features [1,2,3,4]. The actin-based microfilaments, together with myosins, are primarily responsible for force generation and thereby determine contraction, motility and postmitotic cell separation. The tubulin-based microtubules provide the tracks for directed intracellular transport of cargo including proteins, chromosomes and organelles. The intermediate filaments establish a mechanically resistant scaffold ensuring tissue stability and coherence. They are the most diverse in terms of molecular composition with more than 70 polypeptide subunits identified in human that are expressed in cell type- and context-dependent combinations. While microfilaments and microtubules have been extensively studied because of their essential contribution to many cellular processes that can be investigated in vitro, much less is known about intermediate filaments whose function becomes apparent only in living organisms in many instances. The more than 80 diseases that have been linked to perturbations in intermediate filament proteins attest to their crucial importance in humans [4,5].

**Figure 1:**
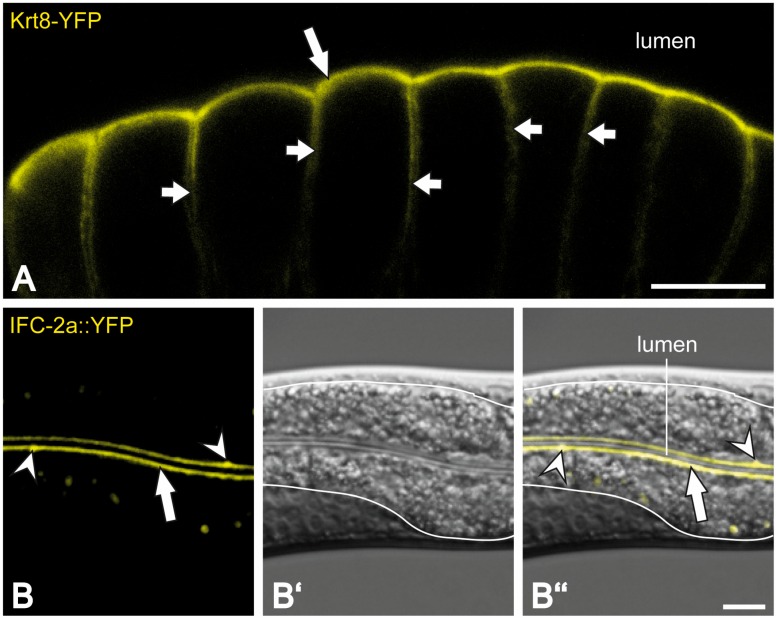
Adluminal enrichment of intermediate filaments is conserved between the mouse and nematode intestine. The micrographs show a comparison of the evolutionary conserved distribution of fluorescently tagged intermediate filament proteins in intestinal cells of knock-in mice ((**A**); [10]) and *C. elegans* (**B**); (**A**) The fluorescent keratin 8 reporter Krt8-YFP is enriched below the apical adluminal membrane domain in murine enterocytes (big arrow). Additional weaker fluorescence is also detectable underneath lateral membranes of adjacent cells (small arrows); (**B**–**B''**) The fluorescence micrograph in (**B**) (corresponding differential contrast image in (**B'**) and merged images in (**B''**)) depicts the fluorescent IFC-2 reporter IFC-2a::YFP in the intestine of *C. elegans*. It is almost exclusively localized to the adluminal membrane domain (arrow). Note the local enrichment at cell-cell junctions of neighboring cells (arrowheads). In addition, unrelated autofluorescent granula are occasionally seen in the cytoplasm. The white lines in (**B'**) and (**B''**) mark the basal border of the intestinal cells. Scale bars = 10 μm.

**Figure 2:**
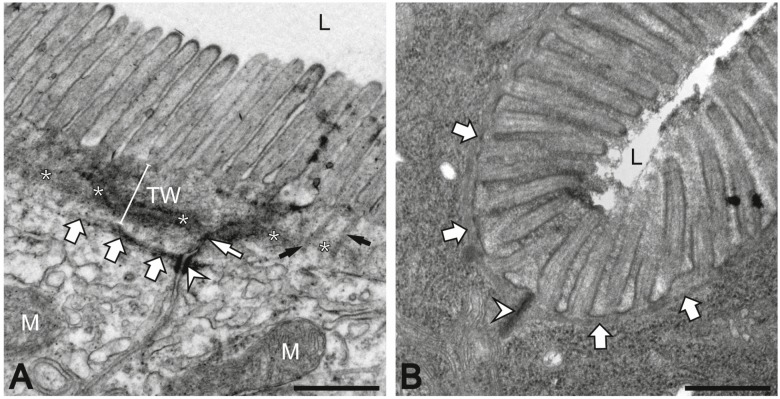
The intermediate filament network of intestinal cells in *Mus musculus* and *Caenorhabditis elegans* is concentrated below the terminal web and is attached to the apical junction complex. The images show electron micrographs of the apical domain of adjacent cells in the murine small intestine (**A**) and the intestine of wild type *C. elegans* (**B**); (**A**) Note the regular arrangement of the apical microvilli next to the lumen (L) containing parallel actin filament bundles that are anchored through distinct rootlets (black arrows) to the terminal web region (TW). The belt of juxtamembraneous actin that is attached to adherens junctions (thin white arrow) is marked by (*). Intermediate filament bundles (thick white arrows) are positioned below the terminal web and connected to desmosomes (arrowhead); (**B**) The corresponding section from the *C. elegans* intestine also depicts a typical brush border with regularly spaced microvilli next to the lumen (L). The microvillar parallel actin bundles extend into the very narrow apical organelle-free terminal web region that is adjacent to the prominent electron dense and intermediate filament-rich endotube (arrows). The endotube is attached to the *C. elegans* apical junction (CeAJ) at the interface of two neighboring cells (arrowhead). M, mitochondrium. Scale bars = 500 nm.

Intermediate filaments are particularly abundant in epithelial cells. It has been suggested that they function as a protective barrier shielding the organism from various types of environmental challenges and insults [6,7,8,9]. This property is reflected by cell type-specific distribution patterns and molecular interactions invoking multiple pathways. The current review is inspired by the unique subapical enrichment of intermediate filaments in the simple, one-layered epithelium of the intestine that has been conserved from *C. elegans* to human (Figure 1 and Figure 2). By contrasting the situation in mammals with that encountered in *C. elegans* we will highlight basic features of the intermediate filament system in the intestine. We will further consider the question to which degree and how the intermediate filament cytoskeleton below the adluminal membrane contributes to the establishment and maintenance of polarization in the intestinal epithelium.

## 2. The Intermediate Filament Cytoskeleton of the Intestinal Epithelium Is Characterized by Cell Type-Specific Polypeptide Subunits

Detailed reviews on simple epithelial keratins and associated diseases have been published in the past and are recommended for the interested reader [11,12,13,14]. In mammals, intestinal epithelia are characterized by expression of a distinct subset of keratin polypeptides. They include the “simple” type II keratin K8 and type I keratins K18, K19 and K20. While K18 is predominant in the undifferentiated crypt compartment, K20 is predominantly detectable in the villus [15,16,17,18,19]. K8 and K19 are detectable throughout the epithelium lining of the small intestine and colon [15]. A low level expression of K7 has been identified in crypts of murine small intestine [15,20,21]. Upon loss of K8 in the colon, K7 is upregulated and becomes detectable throughout the entire crypt length [22]. Reports on weak K23 expression in intestinal mucosa are not conclusive [23,24], although its upregulation in certain carcinomas, together with K7, has been convincingly demonstrated [23]. Finally, transcripts of keratin K24 were detected in human colon but not in the small intestine [25].

The simple keratins are grouped together with the other keratins into the intermediate filament assembly group 1 (details in [26,27]). Type I keratins co-polymerize with type II keratins to form heterodimers, albeit with somewhat differing affinities depending on the specific pairing [28]. These parallel heterodimers are held together by strong hydrophobic interactions between the rod domains forming extremely stable coiled-coils. The heterodimers then associate antiparallel into tetramers, the non-polar building unit of intermediate filaments. The subsequent stages of tetramer assembly into mature intermediate filaments are only in part understood. First, ~8 tetramers associate laterally to form the ~60 nm unit length filament. Unit length filaments then attach longitudinally resulting in elongating 10 nm intermediate filaments. Even less is known about the mechanisms responsible for cell type-specific and subunit-dependent filament bundling, network formation and subcellular arrangement (for different network morphologies in polarized epithelial cells, see [29]).

The intestine of the nematode *Caenorhabditis elegans* is a one-layered, simple epithelium, which consists of only 20 cells. These cells are derived from the *E* cell, a precursor cell that is formed in the 8-cell stage embryo [30,31,32]. The polarized intestinal cells of the worm surround the lumen as 9 rings. The first ring (*int-1*) is composed of 4 cells whereas the other 8 rings (*int-2* to *int-9*) are made up of 2 cells each. During developmental twisting and staggering the connecting cell-cell junctions become arranged in a ladder-type pattern (cf. [9]). Similar to mammals, *C. elegans* also developed a specialized intestinal subset of intermediate filament polypeptides [33,34]. Six of the 11 cytoplasmic intermediate filament-encoding genes are primarily expressed in the intestine (Table 1; review in [35]). The respective polypeptides are denoted as IFB-2, IFC-1, IFC-2, IFD-1, IFD-2 and IFP-1 (previously referred to as IFE-1). RNAi-mediated knockdown of single intermediate filament polypeptides did not lead to developmental defects and induced only minor structural and functional deficiencies under standard conditions (Table 1; [36,37]). However, when RNAi was performed at 25 °C instead of 20 °C a low degree of embryonic lethality was observed in animals treated with either *ifc-1(RNAi)* or *ifd-2(RNAi)* (Table 1; [36]). Adult lethality was noted in a small percentage of worms treated with *ifc-2(RNAi)* independent of the temperature (Table 1; [36]). A possible reason for the overall rather mild phenotypes and their low penetrance may be the redundancy of the intestinal intermediate filament polypeptides. This redundancy may be a major evolutionary advantage for the survival of the worm, which relies on constant uptake of nutrients from its natural environment containing a multitude of harmful substances and microorganisms.

**Figure 3:**
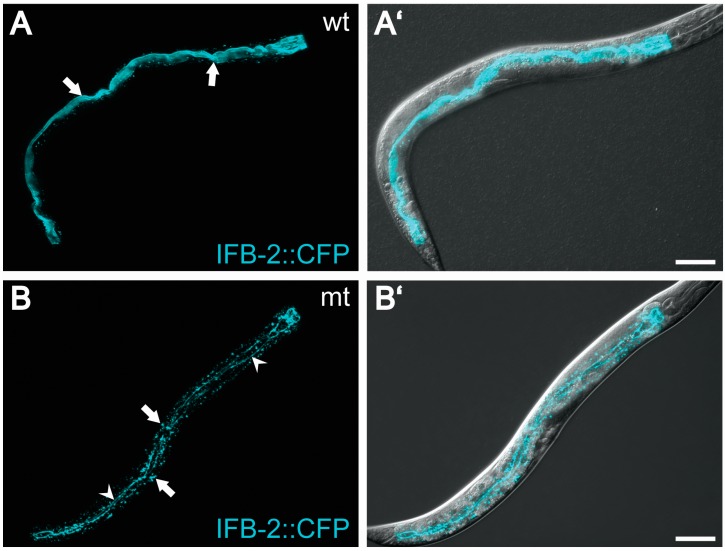
Mutation of the intestinal filament organizer IFO-1 leads to a collapse of the intermediate filament network onto the *C. elegans* apical junction (CeAJ). (**A**) The confocal fluorescence micrographs (projection views) show the distribution of the fluorescent transgene product IFB-2: CFP in a wild type background (wt; strain BJ49; see [58]); Note the exclusive fluorescence in the intestine (anterior to the right upper corner; overlay image with Nomarski optics in (**A**')); IFB-2: CFP is concentrated in the evenly shaped endotube (arrows), which demarcates the ovoid intestinal lumen; (**B**) shows the distribution of IFB-2: CFP in *ifo-1* mutant strain BJ133 (mt; [58]); Note that the intermediate filament network of the endotube has collapsed completely into aggregates decorating the *C. elegans* apical junction (fine lines marked by arrowheads) and formed cytoplasmatic granules (arrows). Overlay image with Nomarski optics in (**B**'). Scale bars = 50 μm.

Pioneering work by Karabinos et al. [38] revealed the heteropolymeric nature of the non-intestinal intermediate filaments in *C. elegans*, which is reminiscent of the situation of the keratin polypeptides in mammals and is also found in *Branchiostoma* intestinal intermediate filaments [39]. Karabinos et al. [38] further demonstrated that recombinant IFB-1 binds strongly to IFA-1, IFA-2, IFA-3 and IFA-4 and weakly to IFC-2 and IFP-1 in blot overlay assays. They also showed that IFB-1 forms typical intermediate filaments with IFA-1, IFA-2 and IFA-3 in vitro as assessed by electron microscopy of negative-stained specimen. In support, IFB-1 is always co-expressed with at least one IFA polypeptide in different cell types of *C. elegans* [38]. Presently, it is not known, whether the intestinal intermediate filaments of *C. elegans* are also heteropolymers and if so, which combinations are favorable for filament formation. It has been suggested, however, that IFC-1 and IFC-2 represent a subgroup, which localizes preferentially to the junction complexes in the intestine and pharynx presumably acting as specific linkers to the rest of the cytoplasmic intermediate filament network [36,40]. All other intestinal intermediate filament polypeptides, i.e., IFB-2, IFD-1, IFD-2 and IFP-1, are localized primarily if not exclusively to a region below the apical plasma membrane surrounding the entire intestinal lumen as a dense network (see Figure 1 and Figure 3; [36,37,41,42]; own unpublished observations).

## 3. Intermediate Filaments Are Needed for the Integrity and Function of Intestinal Epithelial Cells

To understand the contribution of keratins to intestinal physiology, several transgenic mouse models have been established (Table 2). Important for the interpretation of some of the resulting complex phenotypes that will be described below is the distribution of endogenous keratins. For example, a loss of K8 can be compensated for by the presence of K7 in enterocytes [20,22]. Similarly, K18 can compensate for the loss of all other type I keratins [44,45]. Conversely, the selective loss of K18 is compensated for by the other type I keratins [46]. Furthermore, dominant negative phenotypes can only be interpreted on the basis of the quantity and isotype of endogenous keratins [15,47,48].

Depending on the genetic background, absence of K8 leads to embryonal lethality. In a C57BL/6x129Sv background the large majority of embryos died in utero during midterm because of internal bleeding with abnormal accumulation of erythrocytes in fetal livers [53]. In contrast, the large majority of keratin 8 null mutants developed in an FVB/N background [20]. 81% of these animals presented anal prolapse and colorectal hyperplasia with diarrhea and inflammation. It was subsequently found out that the spontaneously-developing chronic colitis in KRT8^−/−^ mice went along with an increase of CD4-positive helper type 2 T cells. Interestingly, the colitis was rescueable by treatment with the antibiotics vancomycin and imipenem [50]. Moreover, KRT8^−/−^ colonic enterocytes are more resistant to apoptosis than their wild type counterparts. This phenotype is also reversed by antibiotic treatment, indicating a microflora-dependent mechanism [51]. The absence of keratins may result in sensitization of the immune system to antigens that are otherwise localized in the intestinal lumen [54,55]. In support, loss of keratins has been implicated in compromised intestinal barrier function [56]. Furthermore, it has been reported that interleukins, notably IL-6, induce phosphorylation of S431 in K8 which results in altered intestinal permeability [57]. These observations have led to the suggestion that keratin network alterations may contribute to inflammatory bowel disease in human [56]. In accordance, the K8 mutations G62C, I63V and K464N are associated with intestinal bowel disease [14]. It was further demonstrated that these mutant keratins have discrete deficiencies in assembly [11].

The function of the intestinal intermediate filaments in *C. elegans* is only very little understood. This is in part due to the above-mentioned compositional redundancy. RNAi-mediated knockdown experiments, however, suggest that the different intermediate filament polypeptides contribute to different degrees to the integrity of intestinal cells. Thus, cytoplasmic invaginations were found in IFC-2-depleted intestine but not upon depletion of other intestinal intermediate filament polypeptides [37]. This phenotype was interpreted as a result of a weakened subapical intermediate filament system. Similarly, pronounced alterations of the intestinal lumen developed in mutants, in which the intermediate filament network was completely disrupted (Figure 3; [58]).

## 4. The Polarized Distribution of Intermediate Filaments in Intestinal Cells Is Evolutionarily Conserved

Polarity is a prominent feature of simple epithelial cells [59,60]. It is characterized by the alignment of the apical membrane towards the external environment and of the basal membrane towards the basement membrane connecting it to the interior of the organism. The surface of most cells lining the intestinal lumen is characterized by a brush border (Figure 2). The brush border consists of regularly-spaced and evenly-shaped microvilli that are anchored to the apical cytoplasm. Up to 3000 of these ~1 μm long and 0.1 μm wide apical membrane protrusions make up the enlarged cell surface of individual enterocytes through which nutrient and metabolite exchange occurs. Microvilli contain a core of membrane-attached longitudinal actin filament bundles whose rootlets extend into the apical organelle-free cytoplasmic terminal web region. They are connected to each other and rest on a dense intermediate filament-rich network. This highly-specific arrangement has attracted morphologists who characterized the different types of filament structures and their cross connectivity [61,62]. Later, molecular components were described and localized (e.g., [62,63,64]). Yet, very little is known about the morphogenesis and maintenance of this complex scaffold with its associated membrane domains and the contribution of individual components to intestinal functions. These functions rely on a selective barrier, which favors nutrient flux, regulates ion and water movements, and limits host contact with the massive intraluminal load of dietary antigens and microbes. Disruption of the barrier contributes to the pathogenesis of a spectrum of human diseases, including food allergy and inflammatory bowel diseases, and may be related to autoimmune diseases and metabolic syndrome [65]. Here, we will focus on the intermediate filament system that is strategically located between the terminal web and the remainder of the intestinal cytoplasm and has received comparatively little attention in the past but may be important for enterocyte organization and function.

Keratin intermediate filaments of enterocytes are subapically enriched in a dense filamentous network just below the terminal web region (Figure 1 and Figure 2; [16,17,66]). They are anchored to desmosomal cell-cell contacts and have been referred to as “desmosomal web” or “apical skeletal disc” [66,67,68,69]. The mutual interdependency of keratins and desmosomes has been noted in many physiological and pathological situations [70,71,72,73].

A comparable arrangement of the intestinal intermediate filament cytoskeleton is encountered in many organisms including also those with a much simpler body plan such as the hexapod *Isotomurus maculatus* [74] and the nematode *Caenorhabditis elegans* (Figure 1 and Figure 2). At the ultrastructural level, the characteristic brush border in *C. elegans* is very similar to that found in mammals. But the intermediate filaments are particularly abundant and are a major and essential component of the nematode-specific endotube (arrows in Figure 2B; [41,75]). The endotube is an electron-dense structure below the comparatively narrow and organelle-free terminal web. It is composed of a dense filamentous mesh and extends throughout the entire intestine, connecting the junctional complexes attaching neighboring cells (arrowheads in Figure 1B–B'' and 2B; see also [9,35,37,41,58]).

## 5. Intermediate Filaments Are Anchored to the Apical Junction Complex in the Intestinal Epithelium

In the mammalian intestinal epithelium keratins are anchored to desmosomes that are positioned below the most apical tight junction and the actin-anchoring adherens junction (Figure 2A). Together, these junctions form the tripartite apical junction complex that regulates paracellular tightness, membrane domain polarization and cytoskeletal organization. The linkage of intermediate filaments to the desmosomal adhesive cadherins is mediated through distinct linker molecules, notably plakin repeat domain-containing desmoplakins and armadillo repeat-containing plakophilins and plakoglobin [72,76]. Disruption of this anchorage in intestine-specific desmoplakin knockouts did not affect apical junction formation and, even more importantly, did not perturb apical keratin filament deposition [77]. Instead and quite unexpected, reduced microvillus length was detected in these animals underscoring the importance of an intact keratin-desmosome scaffold for proper brush border organization [77]. This finding also provides evidence that non-desmosomal factors are responsible for apical keratin localization.

The compact *C. elegans* apical junction (CeAJ; arrowhead in Figure 2B) is the equivalent of the mammalian tripartite junctional complex (detailed review in [32,78,79]). This homogenous electron dense structure, however, is divided into subdomains with unique molecular composition. These include the most apical part presumably encompassing tight junction proteins [80] followed by the cadherin-catenin complex [81] and the most basal DLG-1/AJM-1 complex [82,83,84]. Interestingly, none of the interfering RNAs against CeAJ components caused the intermediate filament network to detach from the junction, while knockdown of the basolaterally localized *C. elegans* Scribble homologue *let-413* led to a spreading of junctional components together with intermediate filaments along the basolateral membrane domain [41,84,85]. An intermediate filament-anchoring function of the DLG-1/AJM-1 complex was indirectly suggested by Carberry et al. [58]. They reported that *dlg-1(RNAi)* released intermediate filament aggregates that had collapsed onto the CeAJ in a mutant strain. In contrast, downregulation of components of the cadherin-catenin complex did not release the intermediate filament aggregates from the CeAJ [58].

## 6. Intermediate Filaments Are Linked to the Actin and Microtubule Cytoskeleton in Intestinal Cells

Early on, morphological evidence was presented for a direct linkage between keratin filaments and microvillar rootlets in the terminal web region. Hirokawa et al. [61] presented stunning electronmicroscopic images of intestinal cells that had been prepared by the quick-freeze, deep-etch, and rotary-replication method. Thin fibrils were identified connecting the striated actin filaments and intermediate filaments that were best detected after extraction with buffers containing 0.3 M KCl and 1 mM ATP which removes myosin fibrils and other terminal web components. The precise nature of these connecting filaments has not been elucidated to date, although initial observations implicated fodrin as a potential candidate [61]. Another candidate linker molecule is the actin bundling protein plastin 1 that has been identified in a more recent study as an important and direct molecular link between microvillar actin rootlets and the keratin cytoskeleton [86]. Plastin-1 knockout mice presented a very fragile brush border with shorter microvilli that had a constricted base and lacked rootlets. Functionally, reduced transepithelial resistance and increased sensitivity to dextran sodium sulfate-induced colitis were observed [86]. Experimental evidence has also been presented for the cross talk between apical actin and keratin filaments. Thus, downregulation of K19 in cultured colon carcinoma cells resulted in disorganization of actin filaments [87] and local disturbances in the formation of microvilli [21]. Partial loss of F-actin was also observed in colonocytes of keratin 8 knockout mice [49].

Five actin genes are expressed in *C. elegans*. ACT-5 is exclusively expressed in the intestinal brush border, where it forms parallel bundles in the microvillus that extend through the terminal web all the way to the endotube (Figure 2B; [42]). It has been shown that ACT-5 is indispensible for correct microvilli formation [42]. It is presently not clear, however, how the microvillar actin is attached to the endotube. Potential candidates are VAB-10, an orthologue of the multifunctional cytoskeletal cross linker plectin [88], and plastin orthologues. Another interesting candidate is the intestinal filament organizer IFO-1 that was identified in a mutagenesis screen of worms carrying a fluorescent IFB-2 reporter [58]. Loss of IFO-1 resulted in disruption of the subapical intermediate filament-rich endotube. The intermediate filament polypeptides accumulated instead in large aggregates next to the CeAJ and in the cytoplasm (Figure 3). The *ifo-1* mutants developed slower, were smaller, produced less progeny and had a reduced life span. Most importantly, apical actin was significantly reduced as determined by phalloidin staining and anti-actin staining. In contrast to the ACT-5-depleted worms [42], the IFO-1-deficient worms still presented a brush border consisting of intact microvilli, although they were slightly disordered. The reason for this comparatively mild phenotype may be the continued presence of substantial amounts of ACT-5 while the apical localization of other actin isoforms, that are not essential for microvillus formation, may have been more severely affected. The observation of concurrent loss of apical intermediate filament polypeptides and actin in *ifo-1* mutants can be taken as an indication for an IFO-1-mediated intermediate filament-actin cross talk. Interestingly, depletion of the ezrin-radixin-moesin orthologue ERM-1, which is known to tether actin to the plasma membrane [89,90], led to a reduction of both apical actin and intermediate filaments which was exacerbated in the *ifo-1* mutant background [58].

It has been shown that downregulation of K19 in cultured colon carcinoma cells induces disorganized microtubules [87]. Later on, γ-tubulin was identified as a potential link between the keratin and microtubule systems. γ-tubulin localized to the apical keratin-rich domain as shown by immunohistology, and co-immunoprecipitated with keratins [91]. Furthermore, K19 downregulation resulted in redistribution of γ-tubulin. Mechanistically, the interaction between keratins and the γ-tubulin ring complex can be disrupted by phosphorylation of GCP6 through a cyclin dependent kinase [92]. Moreover, analysis of small intestinal enterocytes in K8 null mice revealed scattered γ-tubulin in the cytoplasm and disorganized microtubules [21]. A connection between the microtubule-associated proteins ninein, Lis1, Ndel1 and CLIP170 and desmosomes has been described [93,94,95,96], which may be important for the cross talk between the microtubule and keratin-desmosome systems. Presumably, desmoplakin recruits these proteins to the cell cortex to support cortical microtubule localization in suprabasal cells of the epidermis [95]. On the other hand, the microtubule distribution is not dependent on desmoplakin in the intestine, although desmoplakin is still required to recruit ninein and Lis1 to the apical domain [77]. Perhaps, Ndel1 is responsible for keratin transport and/or promotion of local keratin assembly in the intestine, since it has been shown to be able to bind to intermediate filaments [97].

The relationship between microtubules and intermediate filaments has not been investigated in detail in the *C. elegans* intestine. It is known, however, that microtubules become concentrated above the nucleus during organogenesis [31]. They are oriented with their minus ends to the adluminal membrane where γ-tubulin is located [98,99]. Furthermore, orthologues of the microtubule-binding proteins Ndel1, ninein and Lis1 may perform important functions for the cross-talk between junctions, intermediate filaments and microtubules in the *C. elegans* intestine [99,100].

## 7. Intermediate Filaments Affect the Distribution of Membrane Proteins in Polarized Epithelia

Downregulation of K19 in cultured colon carcinoma cells was shown to result in redistribution and depletion of apical membrane proteins [87]. This was further supported by observations in keratin-mutant mice. In enterocytes of the small intestine of K8 null mice a drastic reduction of apical syntaxin 3 and increased intracellular localization of the apical membrane components cystic fibrosis transmembrane regulator (CFTR), sucrase isomaltase and alkaline phosphatase were reported [21]. Examination of enterocytes of the same mouse strain revealed partial loss of H^+^, K^+^-ATPase and basolateral redistribution of the anion exchanger AE1/2 and the Na-transporter EnaC-γ [49]. Although colonic conductance was not significantly altered, diarrhea and higher stool water content was noted in Krt8^−/−^ mice and reduced Na^+^ absorption with reversal of net Cl^−^ movement. Further findings indicated a role of K8 in regulating the apical localization of the glucose transporters 1 and 3 in embryonic epithelia, which was shown to affect mTOR signaling [101]. An interesting finding is the identification of the keratin-binding protein Albatross, which complexes with PAR-3 and regulates the formation of the apical apical junction complex in polarized epithelial cells [102].

In analogy to the observations in mouse, one would expect perturbation of the distribution of apical membrane proteins in the intestine of *C. elegans ifo-1* mutants. This expectation is supported by the developmental and growth defects in *ifo-1*-mutant worms which likely reflect compromised uptake of nutrients. This may be caused by depletion of apically-enriched proteins that are involved in digestion and nutrient uptake. Multiple factors may contribute to this reduction: (i) The overall surface area of the disordered microvilli may be reduced; (ii) The positioning of the microtubule minus ends may be perturbed resulting in less efficient polarized trafficking; (iii) The absence of the endotube may lead to a reduced apico-basal cytoplasmic compartmentalization. These hypotheses have not been examined in detail to date. So far, selective downregulation of the intestinal intermediate filament polypeptide IFC-2 did not affect the localization of the peptide transporter PEPT-1 in the adluminal membrane [37].

## 8. Intermediate Filaments Interact with the PAR-aPKC Polarity Complex in Polarized Epithelia

Cell polarity has been shown to rely on and be determined by the evolutionary conserved PAR-aPKC system [60,103]. At its center is a complex consisting of atypical protein kinase C (aPKC) and the partition-defective (PAR) PDZ domain-containing scaffold proteins PAR-3 and PAR-6. In mammals, this complex has been localized to the apical membrane of rat intestinal epithelia in close proximity to tight junctions [104]. Mashukova et al. [105] showed that active aPKCι and chaperone Hsp70.1 associate with filamentous keratins. They further presented evidence that the keratin network provides a selective scaffold for multiple kinases. It was demonstrated that filamentous keratin and Hsp70 are required for rescue phosphorylation of mature aPKC which is responsible for regeneration of the enzymatic activity [105]. A consequence of the apical keratin-dependent aPKC activation may be activation of the cortical actin-binding protein ezrin [90]. It was shown that keratin filaments recruit dormant, i.e., non-phosphorylated ezrin, which becomes phosphorylated at T567 by aPKCι in the apical domain [106,107]. This may be a key mechanism for organizing the apical juxtamembraneous cytoskeleton.

The pioneering work of Leung et al. [31] revealed the first steps in polarization of the developing *C. elegans* intestine. This process involves rotation of the centrosomes towards the midline and the migration of nuclei towards the apical cell pole [31]. Even before a lumen is formed, the junctional proteins are enriched at the apical plasma membrane together with associated cytoskeletal elements [31,58,108,109,110,111]. Notably, the apical enrichment of IFO-1 precedes slightly IFB-2 and can be detected in early morphogenesis of the intestinal cells [58]. It is not clear, however, whether the apical localization of IFO-1 and IFB-2 depends on actin filaments or microtubules. It is noteworthy, that IFB-2 is still recruited to the apical junction in the *ifo-1*-mutant background, although a contiguous apical network is not established any more [58]. The interaction between intermediate filaments, PKC and ezrin, which has been characterized in mouse (see above), may also be relevant for *C. elegans*. All three partners are located at the apical plasma membrane [31,41,108]. Furthermore, *ifo-1* and *erm-1* mutations are synergistic in intestinal cell adhesion [58,112]. It is attractive to speculate that the function of ERM in organizing the apical cytoskeleton is regulated by a PKC-mediated phosphorylation mechanism in analogy to the situation in mammals. Remarkably, the phosphoepitope of ERM is conserved from human to *C. elegans* [112].

## 9. Conclusions

One might ask whether the distribution of the intermediate filament network in epithelial cells is simply a consequence of polarity or whether intermediate filaments by themselves influence the process of polarization. Although the non-polarized nature of intermediate filament proteins renders them unable to “point” into a direction, it has been shown that intermediate filaments can indeed have an impact on cell polarity.

Interestingly, the antibody IFA which was raised against a highly conserved motif in various intermediate filament proteins [113] was used to identify centrosomes in the *C. elegans* intestine [31]. This observation suggests that an intermediate filament protein may be a centrosomal component. The IFA antibody revealed apical positioning of the centrosomes and formation of an asymmetric microtubule network to be one of the first events during the polarization of *C. elegans* intestinal cells. The apically enriched microtubule network is obviously retained in the adult intestine, since γ-tubulin, which is a component of the γ-tubulin ring complex, promotes the nucleation of microtubules [114], remains localized at the apical membrane [98]. The same positioning of γ-tubulin was found in human colon adenocarcinoma-derived CACO-2 cells, indicating that apical localization of the microtubule organizing centers is evolutionary conserved in polarized epithelia [91]. Moreover, this localization is dependent on GCP6, which in turn binds to keratin filaments [92]. Furthermore, the observed disorganization of the microtubule network after downregulation of K19 and K8 indicates a striking contribution of intermediate filaments to the proper organization of microtubules [21,87]. On the other hand, microtubules are involved in transport of intermediate filament particles and may thereby affect polarized intermediate filament network formation [115].

Additionally to γ-tubulin, keratin filaments co-localize in the apical domain of mammalian intestinal cells with F-actin [116]. In contrast to common belief, polarization of keratin filaments may precede polarization of actin filaments. In cultured CACO-2 cells intermediate filaments are deposited apically at day 3 after seeding, whereas actin accumulates in the developing brush border only after day 5 [106]. Similarly, distribution of keratin filaments along the crypt-villus axis is consistently apically, whereas the brush border differentiates only fully, when cells exit crypts and move toward villi in the small intestine [116]. Moreover, in *C. elegans* the endotube is still maintained in the absence of ACT-5 and in *cct-5* mutants, in which the apical actin cytoskeleton is almost completely lost [42,117]. On the other hand, disruption of the endotube in *ifo-1*-mutant animals leads to drastic reduction in apical actin [58]. Taken together, these observations argue for a dominant function of intermediate filaments with respect to the actin-based cytoskeleton. Intermediate filaments may provide a structural scaffold, which anchors factors that promote actin polymerization. Potential candidates are cordon-bleu and syndapin 2 [118,119]. The intestinal intermediate filament network may also act as a counter bearing against the force of protruding actin filament bundles during microvillar growth and dynamics [120,121]. In support, rebuilding of the apical membrane domain in enterocytes after destruction of the brush border by high pressure involves an accumulation of fibrillary material, that may be composed of intermediate filaments, at the terminal web region [121].

**Figure 4:**
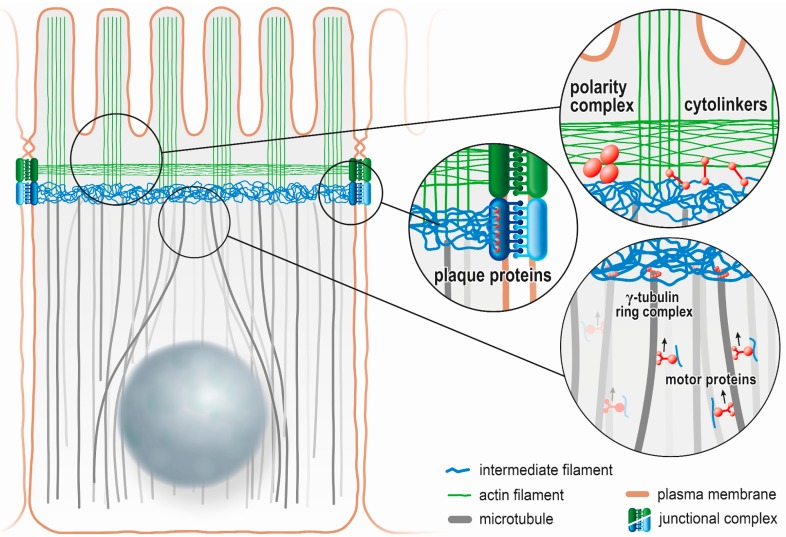
The scheme summarizes the role of intermediate filaments in the establishment and maintenance of polarity in intestinal cells as detailed in the text.

Taken together, one may envision that microtubules form the tracks, along which intermediate filaments are transported to the apical membrane domain, where they provide a construction platform, above which the actin cytoskeleton and its associated specialized membrane structures assemble (summary in Figure 4). It should be kept in mind however, that the cytoskeletal self-organizing principles are under the influence of master regulators. For example, when intestinal polarity is abrogated by *let-413* downregulation in *C. elegans,* the intermediate filament network shows the same redistribution as the junctional proteins along the basolateral membrane [41]. This clearly demonstrates that regulators of cell polarity are the primary determinants of intermediate filament network polarization.

## Figures and Tables

**Table 1:** Distribution and RNAi phenotypes of intestinal intermediate filament proteins in *C. elegans*.

Intermediate Filament Protein	Distribution	RNAi Phenotype				Reference
		Feeding		Microinjection		
		20 °C	25 °C	20 °C	25 °C	
IFB-2	intestine	n.d.	n.d.	n.d.	n.d.	[36,37,41,43]
IFC-1	intestine, hypodermis, pharyngeal junctions of early larvae	n.d.	early larval arrest (10%), abnormal epidermal morphology mostly in head region, occasional muscle detachment defects	n.d.	late embryonic lethal (7%), early larval arrest (16%), abnormal epidermal morphology mostly in head region, occasional muscle detachment defects	[36]
IFC-2	intestine	adult lethal (3%)	adult lethal (10%), ruptured vulva/anus	dumpy (10%)	adult lethal (16%), ruptured vulva/anus	[36]
		luminal invaginations into cytoplasm of intestinal cells				[37]
IFD-1	intestine	n.d.	n.d.	n.d.	n.d.	[36]
IFD-2	intestine	n.d.	n.d.	n.d.	late embryonic lethal (10%), early larval arrest (3%), morphological defects	[36]
IFP-1 (formerly IFE-1)	intestine	n.d.	n.d.	n.d.	n.d.	[36]

n.d. = no phenotype detected.

**Table 2:** Intestinal phenotypes of murine keratin mutants (n.d. = no phenotype detected).

Transgenesis	Intestinal Phenotype	Reference
KRT8^−/−^	colorectal hyperplasia	[20]
	scattered γ-tubulin, disorganized microtubules, reduced apical syntaxin, aberrant intracellular localization of CFTR, sucrase isomaltase, alkaline phosphatase	[21]
	diarrhea, reduced apical F-actin, partial loss of H^+^, K^+^-ATPase and basolateral redistribution of the anion exchanger AE1/2 and the Na-transporter EnaC-γ	[49]
	spontaneous chronic T helper type 2 colitis	[50]
	microflora-dependent resistance to apoptosis	[51]
KRT8^+/−^	increased crypt length, increased sensitivity to experimental colitis	[22]
KRT7^−/−^	n.d.	[52]
KRT18^−/−^	n.d.	[46]
KRT19^−/−^	n.d.	[45]
keratin type I^−/−^ (except KRT18)	n.d.	[44]
hK18 R89C	partial disruption of keratin filament network	[48]
	aggregate formation	[47]
hK20 R80H	partial disruption of keratin filament network	[15]

## References

[1] Straube A., Bradshaw R.A., Stahl P.D. (2016). Microtubules and microtubule-associated proteins (MAPs). Encyclopedia of Cell Biology.

[2] Heissler S.M., Sellers J.R., Bradshaw R.A., Stahl P.D. (2016). Myosins. Encyclopedia of Cell Biology.

[3] Pernier J., Montaville P., Carlier M.F., Bradshaw R.A., Stahl P.D. (2016). Actin assembly dynamics and its regulation in motile and morphogenetic processes. Encyclopedia of Cell Biology.

[4] Leube R.E., Schwarz N., Bradshaw R.A., Stahl P.D. (2016). Intermediate filaments. Encyclopedia of Cell Biology.

[5] Omary M.B. (2009). “IF-pathies”: A broad spectrum of intermediate filament-associated diseases. J. Clin. Invest..

[6] Pekny M., Lane E.B. (2007). Intermediate filaments and stress. Exp. Cell Res..

[7] Toivola D.M., Strnad P., Habtezion A., Omary M.B. (2010). Intermediate filaments take the heat as stress proteins. Trends Cell Biol..

[8] Geisler F., Leube R.E. (2016). Epithelial intermediate filaments: Guardians against microbial infection?. Cells.

[9] Jahnel O., Hoffmann B., Merkel R., Bossinger O., Leube R.E. (2016). Mechanical probing of the intermediate filament-rich *Caenorhabditis elegans* intestine. Methods Enzymol..

[10] Schwarz N., Windoffer R., Magin T.M., Leube R.E. (2015). Dissection of keratin network formation, turnover and reorganization in living murine embryos. Sci. Rep..

[11] Owens D.W., Lane E.B. (2003). The quest for the function of simple epithelial keratins. Bioessays.

[12] Omary M.B., Ku N.O., Strnad P., Hanada S. (2009). Toward unraveling the complexity of simple epithelial keratins in human disease. J. Clin. Invest..

[13] Majumdar D., Tiernan J.P., Lobo A.J., Evans C.A., Corfe B.M. (2012). Keratins in colorectal epithelial function and disease. Int. J. Exp. Pathol..

[14] Owens D.W., Lane E.B. (2004). Keratin mutations and intestinal pathology. J. Pathol..

[15] Zhou Q., Toivola D.M., Feng N., Greenberg H.B., Franke W.W., Omary M.B. (2003). Keratin 20 helps maintain intermediate filament organization in intestinal epithelia. Mol. Biol. Cell.

[16] Moll R., Schiller D.L., Franke W.W. (1990). Identification of protein it of the intestinal cytoskeleton as a novel type I cytokeratin with unusual properties and expression patterns. J. Cell Biol..

[17] Moll R., Zimbelmann R., Goldschmidt M.D., Keith M., Laufer J., Kasper M., Koch P.J., Franke W.W. (1993). The human gene encoding cytokeratin 20 and its expression during fetal development and in gastrointestinal carcinomas. Differentiation.

[18] Calnek D., Quaroni A. (1993). Differential localization by in situ hybridization of distinct keratin mRNA species during intestinal epithelial cell development and differentiation. Differentiation.

[19] Quaroni A., Calnek D., Quaroni E., Chandler J.S. (1991). Keratin expression in rat intestinal crypt and villus cells. Analysis with a panel of monoclonal antibodies. J. Biol. Chem..

[20] Baribault H., Penner J., Iozzo R.V., Wilson-Heiner M. (1994). Colorectal hyperplasia and inflammation in keratin 8-deficient FVB/N mice. Genes Dev..

[21] Ameen N.A., Figueroa Y., Salas P.J. (2001). Anomalous apical plasma membrane phenotype in CK8-deficient mice indicates a novel role for intermediate filaments in the polarization of simple epithelia. J. Cell Sci..

[22] Asghar M.N., Silvander J.S., Helenius T.O., Lahdeniemi I.A., Alam C., Fortelius L.E., Holmsten R.O., Toivola D.M. (2015). The amount of keratins matters for stress protection of the colonic epithelium. PLoS ONE.

[23] Birkenkamp-Demtroder K., Mansilla F., Sorensen F.B., Kruhoffer M., Cabezon T., Christensen L.L., Aaltonen L.A., Verspaget H.W., Orntoft T.F. (2007). Phosphoprotein keratin 23 accumulates in MSS but not MSI colon cancers in vivo and impacts viability and proliferation in vitro. Mol. Oncol..

[24] Rogers M.A., Winter H., Langbein L., Bleiler R., Schweizer J. (2004). The human type I keratin gene family: Characterization of new hair follicle specific members and evaluation of the chromosome 17q21.2 gene domain. Differentiation.

[25] Sprecher E., Itin P., Whittock N.V., McGrath J.A., Meyer R., DiGiovanna J.J., Bale S.J., Uitto J., Richard G. (2002). Refined mapping of Naegeli-Franceschetti-Jadassohn syndrome to a 6 cM interval on chromosome 17q11.2-q21 and investigation of candidate genes. J. Invest. Dermatol..

[26] Strelkov S.V., Herrmann H., Aebi U. (2003). Molecular architecture of intermediate filaments. Bioessays.

[27] Herrmann H., Hesse M., Reichenzeller M., Aebi U., Magin T.M. (2003). Functional complexity of intermediate filament cytoskeletons: From structure to assembly to gene ablation. Int. Rev. Cytol..

[28] Hofmann I., Franke W.W. (1997). Heterotypic interactions and filament assembly of type I and type II cytokeratins in vitro: Viscometry and determinations of relative affinities. Eur. J. Cell Biol..

[29] Iwatsuki H., Suda M. (2010). Seven kinds of intermediate filament networks in the cytoplasm of polarized cells: Structure and function. Acta Histochem. Cytochem..

[30] Goldstein B. (1993). Establishment of gut fate in the E lineage of *C. elegans*: The roles of lineage-dependent mechanisms and cell interactions. Development.

[31] Leung B., Hermann G.J., Priess J.R. (1999). Organogenesis of the *Caenorhabditis elegans* intestine. Dev. Biol..

[32] McGhee J.D. The *C. elegans* Intestine. http://www.wormbook.org/chapters/www_intestine/intestine.html.

[33] Dodemont H., Riemer D., Ledger N., Weber K. (1994). Eight genes and alternative RNA processing pathways generate an unexpectedly large diversity of cytoplasmic intermediate filament proteins in the nematode *Caenorhabditis elegans*. EMBO J..

[34] Karabinos A., Schmidt H., Harborth J., Schnabel R., Weber K. (2001). Essential roles for four cytoplasmic intermediate filament proteins in *Caenorhabditis elegans* development. Proc. Natl. Acad. Sci. USA.

[35] Carberry K., Wiesenfahrt T., Windoffer R., Bossinger O., Leube R.E. (2009). Intermediate filaments in *Caenorhabditis elegans*. Cell Motil. Cytoskeleton.

[36] Karabinos A., Schunemann J., Weber K. (2004). Most genes encoding cytoplasmic intermediate filament (IF) proteins of the nematode *Caenorhabditis elegans* are required in late embryogenesis. Eur. J. Cell Biol..

[37] Hüsken K., Wiesenfahrt T., Abraham C., Windoffer R., Bossinger O., Leube R.E. (2008). Maintenance of the intestinal tube in *Caenorhabditis elegans*: The role of the intermediate filament protein IFC-2. Differentiation.

[38] Karabinos A., Schulze E., Schunemann J., Parry D.A., Weber K. (2003). In vivo and in vitro evidence that the four essential intermediate filament (IF) proteins A1, A2, A3 and B1 of the nematode *Caenorhabditis elegans* form an obligate heteropolymeric IF system. J. Mol. Biol..

[39] Karabinos A., Schunemann J., Parry D.A., Weber K. (2002). Tissue-specific co-expression and in vitro heteropolymer formation of the two small branchiostoma intermediate filament proteins A3 and B2. J. Mol. Biol..

[40] Karabinos A., Schulze E., Klisch T., Wang J., Weber K. (2002). Expression profiles of the essential intermediate filament (IF) protein A2 and the IF protein C2 in the nematode *Caenorhabditis elegans*. Mech. Dev..

[41] Bossinger O., Fukushige T., Claeys M., Borgonie G., McGhee J.D. (2004). The apical disposition of the *Caenorhabditis elegans* intestinal terminal web is maintained by LET-413. Dev. Biol..

[42] MacQueen A.J., Baggett J.J., Perumov N., Bauer R.A., Januszewski T., Schriefer L., Waddle J.A. (2005). ACT-5 is an essential *Caenorhabditis elegans* actin required for intestinal microvilli formation. Mol. Biol. Cell.

[43] Segbert C., Johnson K., Theres C., van Furden D., Bossinger O. (2004). Molecular and functional analysis of apical junction formation in the gut epithelium of *Caenorhabditis elegans*. Dev. Biol..

[44] Kumar V., Bouameur J.E., Bar J., Rice R.H., Hornig-Do H.T., Roop D.R., Schwarz N., Brodesser S., Thiering S., Leube R.E. (2015). A keratin scaffold regulates epidermal barrier formation, mitochondrial lipid composition, and activity. J. Cell Biol..

[45] Tamai Y., Ishikawa T., Bosl M.R., Mori M., Nozaki M., Baribault H., Oshima R.G., Taketo M.M. (2000). Cytokeratins 8 and 19 in the mouse placental development. J. Cell Biol..

[46] Magin T.M., Schroder R., Leitgeb S., Wanninger F., Zatloukal K., Grund C., Melton D.W. (1998). Lessons from keratin 18 knockout mice: Formation of novel keratin filaments, secondary loss of keratin 7 and accumulation of liver-specific keratin 8-positive aggregates. J. Cell Biol..

[47] Hesse M., Grund C., Herrmann H., Brohl D., Franz T., Omary M.B., Magin T.M. (2007). A mutation of keratin 18 within the coil 1A consensus motif causes widespread keratin aggregation but cell type-restricted lethality in mice. Exp. Cell Res..

[48] Ku N.O., Michie S., Oshima R.G., Omary M.B. (1995). Chronic hepatitis, hepatocyte fragility, and increased soluble phosphoglycokeratins in transgenic mice expressing a keratin 18 conserved arginine mutant. J. Cell Biol..

[49] Toivola D.M., Krishnan S., Binder H.J., Singh S.K., Omary M.B. (2004). Keratins modulate colonocyte electrolyte transport via protein mistargeting. J. Cell Biol..

[50] Habtezion A., Toivola D.M., Butcher E.C., Omary M.B. (2005). Keratin-8-deficient mice develop chronic spontaneous th2 colitis amenable to antibiotic treatment. J. Cell Sci..

[51] Habtezion A., Toivola D.M., Asghar M.N., Kronmal G.S., Brooks J.D., Butcher E.C., Omary M.B. (2011). Absence of keratin 8 confers a paradoxical microflora-dependent resistance to apoptosis in the colon. Proc. Natl. Acad. Sci. USA.

[52] Sandilands A., Smith F.J., Lunny D.P., Campbell L.E., Davidson K.M., MacCallum S.F., Corden L.D., Christie L., Fleming S., Lane E.B. (2013). Generation and characterisation of keratin 7 (K7) knockout mice. PLoS ONE.

[53] Baribault H., Price J., Miyai K., Oshima R.G. (1993). Mid-gestational lethality in mice lacking keratin 8. Genes Dev..

[54] Dignass A.U., Baumgart D.C., Sturm A. (2004). Review article: The aetiopathogenesis of inflammatory bowel disease—Immunology and repair mechanisms. Aliment. Pharmacol. Ther..

[55] Shen L., Turner J.R. (2006). Role of epithelial cells in initiation and propagation of intestinal inflammation. Eliminating the static: Tight junction dynamics exposed. Am. J. Physiol. Gastrointest. Liver Physiol..

[56] Zupancic T., Stojan J., Lane E.B., Komel R., Bedina-Zavec A., Liovic M. (2014). Intestinal cell barrier function in vitro is severely compromised by keratin 8 and 18 mutations identified in patients with inflammatory bowel disease. PLoS ONE.

[57] Wang L., Srinivasan S., Theiss A.L., Merlin D., Sitaraman S.V. (2007). Interleukin-6 induces keratin expression in intestinal epithelial cells: Potential role of keratin-8 in interleukin-6-induced barrier function alterations. J. Biol. Chem..

[58] Carberry K., Wiesenfahrt T., Geisler F., Stocker S., Gerhardus H., Uberbach D., Davis W., Jorgensen E., Leube R.E., Bossinger O. (2012). The novel intestinal filament organizer IFO-1 contributes to epithelial integrity in concert with ERM-1 and DLG-1. Development.

[59] Rodriguez-Boulan E., Nelson W.J. (1989). Morphogenesis of the polarized epithelial cell phenotype. Science.

[60] Bryant D.M., Mostov K.E. (2008). From cells to organs: Building polarized tissue. Nat. Rev. Mol. Cell Biol..

[61] Hirokawa N., Tilney L.G., Fujiwara K., Heuser J.E. (1982). Organization of actin, myosin, and intermediate filaments in the brush border of intestinal epithelial cells. J. Cell Biol..

[62] Bement W.M., Mooseker M.S., John E.H., Ian F.P. (1996). The cytoskeleton of the intestinal epithelium: Components, assembly, and dynamic rearrangements. The Cytoskeleton: A Multi-Volume Treatise.

[63] Drenckhahn D., Groschel-Stewart U. (1980). Localization of myosin, actin, and tropomyosin in rat intestinal epithelium: Immunohistochemical studies at the light and electron microscope levels. J. Cell Biol..

[64] Drenckhahn D., Dermietzel R. (1988). Organization of the actin filament cytoskeleton in the intestinal brush border: A quantitative and qualitative immunoelectron microscope study. J. Cell Biol..

[65] Menard S., Cerf-Bensussan N., Heyman M. (2010). Multiple facets of intestinal permeability and epithelial handling of dietary antigens. Mucosal Immunol..

[66] Franke W.W., Appelhans B., Schmid E., Freudenstein C., Osborn M., Weber K. (1979). The organization of cytokeratin filaments in the intestinal epithelium. Eur. J. Cell Biol..

[67] Hemmi A., Komiyama A., Ohno S., Fujii Y., Kawaoi A., Katoh R., Suzuki K. (1995). Different organization of intermediate filaments in columnar cells of rat large intestinal mucosa as revealed by confocal laser scanning microscopy and quick-freezing and deep-etching method. Virchows Arch..

[68] Brown D.T., Anderton B.H., Wylie C.C. (1983). The organization of intermediate filaments in normal human colonic epithelium and colonic carcinoma cells. Int. J. Cancer.

[69] Brunser O., Luft H.J. (1970). Fine structure of the apex of absorptive cell from rat small intestine. J. Ultrastruct. Res..

[70] Gallicano G.I., Kouklis P., Bauer C., Yin M., Vasioukhin V., Degenstein L., Fuchs E. (1998). Desmoplakin is required early in development for assembly of desmosomes and cytoskeletal linkage. J. Cell Biol..

[71] Kröger C., Loschke F., Schwarz N., Windoffer R., Leube R.E., Magin T.M. (2013). Keratins control intercellular adhesion involving PKC-α-mediated desmoplakin phosphorylation. J. Cell Biol..

[72] Holthöfer B., Windoffer R., Troyanovsky S., Leube R.E. (2007). Structure and function of desmosomes. Int. Rev. Cytol..

[73] Getsios S., Huen A.C., Green K.J. (2004). Working out the strength and flexibility of desmosomes. Nat. Rev. Mol. Cell Biol..

[74] Mencarelli C., Ciolfi S., Caroti D., Lupetti P., Dallai R. (2011). Isomin: A novel cytoplasmic intermediate filament protein from an arthropod species. BMC Biol..

[75] Munn E.A., Greenwood C.A. (1984). The occurrence of submicrovillar endotube (modified terminal web) and associated structures in the intestinal epithelia of nematodes. Philos. Trans. R. Soc. Lond. B.

[76] Green K.J., Getsios S., Troyanovsky S., Godsel L.M. (2010). Intercellular junction assembly, dynamics, and homeostasis. Cold Spring Harb. Perspect. Biol..

[77] Sumigray K.D., Lechler T. (2012). Desmoplakin controls microvilli length but not cell adhesion or keratin organization in the intestinal epithelium. Mol. Biol. Cell.

[78] Knust E., Bossinger O. (2002). Composition and formation of intercellular junctions in epithelial cells. Science.

[79] Pasti G., Labouesse M. Epithelial Junctions, Cytoskeleton, and Polarity. http://www.wormbook.org/chapters/www_epithelialjunctionsattach.2/epithelialjunctions.html.

[80] Asano A., Asano K., Sasaki H., Furuse M., Tsukita S. (2003). Claudins in *Caenorhabditis elegans*: Their distribution and barrier function in the epithelium. Curr. Biol..

[81] Costa M., Raich W., Agbunag C., Leung B., Hardin J., Priess J.R. (1998). A putative catenin-cadherin system mediates morphogenesis of the *Caenorhabditis elegans* embryo. J. Cell Biol..

[82] Köppen M., Simske J.S., Sims P.A., Firestein B.L., Hall D.H., Radice A.D., Rongo C., Hardin J.D. (2001). Cooperative regulation of AJM-1 controls junctional integrity in *Caenorhabditis elegans* epithelia. Nat. Cell Biol..

[83] Bossinger O., Klebes A., Segbert C., Theres C., Knust E. (2001). Zonula adherens formation in *Caenorhabditis elegans* requires DLG-1, the homologue of the drosophila gene discs large. Dev. Biol..

[84] McMahon L., Legouis R., Vonesch J.L., Labouesse M. (2001). Assembly of *C. elegans* apical junctions involves positioning and compaction by LET-413 and protein aggregation by the MAGUK protein DLG-1. J. Cell Sci..

[85] Legouis R., Gansmuller A., Sookhareea S., Bosher J.M., Baillie D.L., Labouesse M. (2000). LET-413 is a basolateral protein required for the assembly of adherens junctions in *Caenorhabditis elegans*. Nat. Cell Biol..

[86] Grimm-Günter E.M., Revenu C., Ramos S., Hurbain I., Smyth N., Ferrary E., Louvard D., Robine S., Rivero F. (2009). Plastin 1 binds to keratin and is required for terminal web assembly in the intestinal epithelium. Mol. Biol. Cell.

[87] Salas P.J., Rodriguez M.L., Viciana A.L., Vega-Salas D.E., Hauri H.P. (1997). The apical submembrane cytoskeleton participates in the organization of the apical pole in epithelial cells. J. Cell Biol..

[88] Bosher J.M., Hahn B.S., Legouis R., Sookhareea S., Weimer R.M., Gansmuller A., Chisholm A.D., Rose A.M., Bessereau J.L., Labouesse M. (2003). The *Caenorhabditis elegans* VAB-10 spectraplakin isoforms protect the epidermis against internal and external forces. J. Cell Biol..

[89] Fievet B., Louvard D., Arpin M. (2007). ERM proteins in epithelial cell organization and functions. Biochim. Biophys. Acta.

[90] Fehon R.G., McClatchey A.I., Bretscher A. (2010). Organizing the cell cortex: The role of ERM proteins. Nat. Rev. Mol. Cell Biol..

[91] Salas P.J. (1999). Insoluble γ-tubulin-containing structures are anchored to the apical network of intermediate filaments in polarized CACO-2 epithelial cells. J. Cell Biol..

[92] Oriolo A.S., Wald F.A., Canessa G., Salas P.J. (2007). GCP6 binds to intermediate filaments: A novel function of keratins in the organization of microtubules in epithelial cells. Mol. Biol. Cell.

[93] Sumigray K.D., Chen H., Lechler T. (2011). Lis1 is essential for cortical microtubule organization and desmosome stability in the epidermis. J. Cell Biol..

[94] Sumigray K.D., Lechler T. (2011). Control of cortical microtubule organization and desmosome stability by centrosomal proteins. Bioarchitecture.

[95] Lechler T., Fuchs E. (2007). Desmoplakin: An unexpected regulator of microtubule organization in the epidermis. J. Cell Biol..

[96] Wacker I.U., Rickard J.E., de Mey J.R., Kreis T.E. (1992). Accumulation of a microtubule-binding protein, pp170, at desmosomal plaques. J. Cell Biol..

[97] Nguyen M.D., Shu T., Sanada K., Lariviere R.C., Tseng H.C., Park S.K., Julien J.P., Tsai L.H. (2004). A NUDEL-dependent mechanism of neurofilament assembly regulates the integrity of CNS neurons. Nat. Cell Biol..

[98] Bobinnec Y., Fukuda M., Nishida E. (2000). Identification and characterization of *Caenorhabditis elegans* γ-tubulin in dividing cells and differentiated tissues. J. Cell Sci..

[99] Quintin S., Gally C., Labouesse M. (2016). Noncentrosomal microtubules in *C. elegans* epithelia. Genesis.

[100] Wang S., Wu D., Quintin S., Green R.A., Cheerambathur D.K., Ochoa S.D., Desai A., Oegema K. (2015). NOCA-1 functions with γ-tubulin and in parallel to patronin to assemble non-centrosomal microtubule arrays in *C. elegans*. Elife.

[101] Vijayaraj P., Kroger C., Reuter U., Windoffer R., Leube R.E., Magin T.M. (2009). Keratins regulate protein biosynthesis through localization of GLUT1 and -3 upstream of AMP kinase and raptor. J. Cell Biol..

[102] Sugimoto M., Inoko A., Shiromizu T., Nakayama M., Zou P., Yonemura S., Hayashi Y., Izawa I., Sasoh M., Uji Y. (2008). The keratin-binding protein albatross regulates polarization of epithelial cells. J. Cell Biol..

[103] Suzuki A., Ohno S. (2006). The PAR-aPKC system: Lessons in polarity. J. Cell Sci..

[104] Izumi Y., Hirose T., Tamai Y., Hirai S., Nagashima Y., Fujimoto T., Tabuse Y., Kemphues K.J., Ohno S. (1998). An atypical PKC directly associates and colocalizes at the epithelial tight junction with ASIP, a mammalian homologue of *Caenorhabditis elegans* polarity protein PAR-3. J. Cell Biol..

[105] Mashukova A., Oriolo A.S., Wald F.A., Casanova M.L., Kroger C., Magin T.M., Omary M.B., Salas P.J. (2009). Rescue of atypical protein kinase C in epithelia by the cytoskeleton and Hsp70 family chaperones. J. Cell Sci..

[106] Wald F.A., Oriolo A.S., Casanova M.L., Salas P.J. (2005). Intermediate filaments interact with dormant ezrin in intestinal epithelial cells. Mol. Biol. Cell.

[107] Wald F.A., Oriolo A.S., Mashukova A., Fregien N.L., Langshaw A.H., Salas P.J. (2008). Atypical protein kinase C (iota) activates ezrin in the apical domain of intestinal epithelial cells. J. Cell Sci..

[108] Van Furden D., Johnson K., Segbert C., Bossinger O. (2004). The *C. elegans* ezrin-radixin-moesin protein ERM-1 is necessary for apical junction remodelling and tubulogenesis in the intestine. Dev. Biol..

[109] Feldman J.L., Priess J.R. (2012). A role for the centrosome and PAR-3 in the hand-off of MTOC function during epithelial polarization. Curr. Biol..

[110] Achilleos A., Wehman A.M., Nance J. (2010). PAR-3 mediates the initial clustering and apical localization of junction and polarity proteins during *C. elegans* intestinal epithelial cell polarization. Development.

[111] Totong R., Achilleos A., Nance J. (2007). PAR-6 is required for junction formation but not apicobasal polarization in *C. elegans* embryonic epithelial cells. Development.

[112] Gloerich M., ten Klooster J.P., Vliem M.J., Koorman T., Zwartkruis F.J., Clevers H., Bos J.L. (2012). Rap2A links intestinal cell polarity to brush border formation. Nat. Cell Biol..

[113] Pruss R.M., Mirsky R., Raff M.C., Thorpe R., Dowding A.J., Anderton B.H. (1981). All classes of intermediate filaments share a common antigenic determinant defined by a monoclonal antibody. Cell.

[114] Oakley B.R., Oakley C.E., Yoon Y., Jung M.K. (1990). γ-Tubulin is a component of the spindle pole body that is essential for microtubule function in *Aspergillus nidulans*. Cell.

[115] Helfand B.T., Chang L., Goldman R.D. (2003). The dynamic and motile properties of intermediate filaments. Annu. Rev. Cell Dev. Biol..

[116] Oriolo A.S., Wald F.A., Ramsauer V.P., Salas P.J. (2007). Intermediate filaments: A role in epithelial polarity. Exp. Cell Res..

[117] Saegusa K., Sato M., Sato K., Nakajima-Shimada J., Harada A. (2014). *Caenorhabditis elegans* chaperonin CCT/TRiC is required for actin and tubulin biogenesis and microvillus formation in intestinal epithelial cells. Mol. Biol. Cell.

[118] Wayt J., Bretscher A. (2014). Cordon bleu serves as a platform at the basal region of microvilli, where it regulates microvillar length through its WH2 domains. Mol. Biol. Cell.

[119] Grega-Larson N.E., Crawley S.W., Erwin A.L., Tyska M.J. (2015). Cordon bleu promotes the assembly of brush border microvilli. Mol. Biol. Cell.

[120] Crawley S.W., Mooseker M.S., Tyska M.J. (2014). Shaping the intestinal brush border. J. Cell Biol..

[121] Tilney L.G., Cardell R.R. (1970). Factors controlling the reassembly of the microvillous border of the small intestine of the salamander. J. Cell Biol..

